# The Search for Quorum Sensing in *Botrytis cinerea*: Regulatory Activity of Its Extracts on Its Development

**DOI:** 10.3390/plants9020168

**Published:** 2020-01-31

**Authors:** Esteban D. Rosero-Hernández, Fernando L. Echeverri

**Affiliations:** Química Orgánica de Productos Naturales, Instituto de Química, Universidad de Antioquia, Medellín Cl. 62 #52–59, Antioquia, Colombia; feche@une.net.co

**Keywords:** *Botrytis cinerea*, quorum sensing, fermentation, extracts, biofilm, pellets, sporulation, 1-phenylethanol, 3-phenylpropanol, phytotoxins

## Abstract

*Botrytis cinerea* is a phytopathogenic fungus that causes large crop and post-harvest losses. Therefore, new and effective strategies are needed to control the disease and to reduce resistance to fungicides. Modulating pathogenicity and virulence by manipulating microbial communication is a promising strategy. This communication mechanism, called *Quorum Sensing* (*QS*), has already been reported in bacteria and yeasts; however, it has not yet been studied in *B. cinerea*. To establish the existence of this biochemical process in *B. cinerea*, we prepared extracts at different growth times (D1-D12), which were applied to fresh cultures of the same fungi. The chemical analysis of the extracts obtained from several fermentations showed different compositions and biological activities. We confirmed the presence of several phytotoxins, as well as compounds 1-phenylethanol and 3-phenylpropanol. Day five extract (0.1%) inhibited conidia germination and elongation of germ tubes, day seven extract (1%) produced the greatest phytotoxic effect in tomato leaves, and day nine extract (0.1%) was a sporulation inhibitor. In contrast, the extracts from days 7, 9, and 12 of fermentation (0.1% and 0.01%) promoted pellet and biofilm formation. Sporulation was slightly induced at 0.01%, while at 0.1% there was a great inhibition. At the highest extract concentrations, a biocidal effect was detected, but at the lowest, we observed a *QS*-like effect, regulating processes such as filamentation, morphogenesis, and pathogenesis. These results of the biological activity and composition of extracts suggest the existence of a *QS*-like mechanism in *B. cinerea*, which could lead to new non-biocidal alternatives for its control through interference in the pathogenicity and virulence mechanisms of the fungi.

## 1. Introduction

*Botrytis cinerea* is a phytopathogenic fungus that parasitizes many commercial and nutritionally valuable plant species; for this reason, it is considered one of the most harmful fungi in the field [1,2,3]. Various fungicides are used for its control, but their effectiveness has been reduced by microbial resistance, and along with increased production costs, they generate chemical residues that threaten human and animal health, as well as the environment [4]. 

One of the most studied mechanisms of microbial communication is quorum sensing (*QS*), widely seen in bacteria and some yeasts and filamentous fungi. Through this pathway, the expressions of genes are coordinated by synthesizing diffusible self-inducing chemical signals [5]. This process aims to induce pathogenicity and virulence, which are initially expressed by increasing mobility, the synthesis of toxins, exopolysaccharides, surfactants, and extracellular enzymes, and, finally, the formation of biofilms [6,7]. In fungi and yeasts, molecules such as farnesol, tyrosol, and phenylethanol, among others, were reported to be auto-inducing molecules of *QS* [8].

The interference of *QS* with specific molecules could be a new non-biocidal control alternative that would not generate microbial resistance. However, whether *QS* communication exists in *B. cinerea* is currently unknown, as is the auto-inducing molecules involved. Therefore, in this work, we analyzed the effect and composition of extracts of *B. cinerea* obtained at different growing times on the development and growth of the same fungus in order to provide preliminary evidence of the existence of the *QS* mechanism.

## 2. Results 

### 2.1. Extracts Preparation and Liquid Chromatography/Mass Spectrometry Analysis

The weight of the extract increased according to the fermentation time, obtaining the maximum amount at day nine of the culture (393.9 mg; Table 1). The composition of extracts was analyzed using ultra-high-performance liquid chromatography-mass spectrometry (UHPLC-MS) to detect some of the most representative metabolites of *B. cinerea* and was classified as phytotoxins as well as molecules reported as being *QS* modulators in fungi and yeast [6].

**Figure 1:**
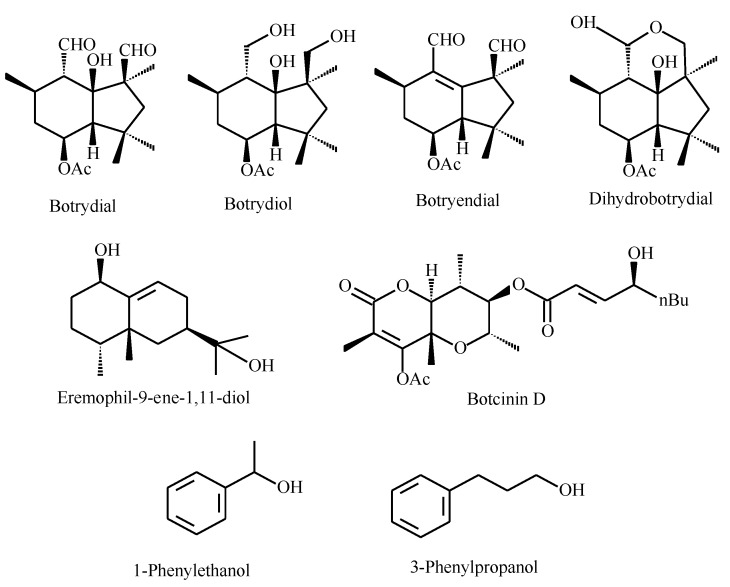
Compounds detected in *B. cinerea* extracts by high-performance liquid chromatography-tandem mass spectrometry (HPLC-MS/MS).

The extracts obtained at days one to seven (D1–D7; Table 2) revealed the presence of some terpenes such as botrydiol (from the D1) and the phytotoxins botrydial and dihydrobotrydial (Figure 1). Botryendial was detected in extracts from D7 to D12, whereas eremophil-9-ene-1,11-diol was noticed in older extracts (D9–D12).

Compounds commonly reported in *QS* in other fungi and yeasts, such as farnesol and tyrosol [9,10,11], were not detected; however, we established the presence of 1-phenylethanol and 3-phenyl-1-propanol in the extracts from D5 to D12 (Table 2).

### 2.2. Effect of Extracts on B. cinerea

A summary of the results obtained from the effect of the extracts on the growth of *B. cinerea* is provided in Table 3.

The inhibitory effect on radial growth was the least important, and the main inhibitory effects on conidia germination, germ tube growth, sporulation, and to a lesser extent, on pellet formation, were especially observed for D1–D9 extracts. The induction of the biofilm occurred with all extracts, as well as a high severity of lesions. With a few exceptions, the older extracts, D9–D12, and even some of the D7 extract, induced several effects, some of them related to pellets, sporulation, and biofilm formation.

In terms of phytotoxicity, the effects of the extracts were compared with that of the botrydial (10 ppm); thus, high lesion incidences with the extracts obtained on D3–D7 and D12 were observed. For severity, we observed a strong effect with D1, D7, and D9 extracts, increasing the effect by up to 218.1% in terms of the affected area and compared to the botrydial control. A more detailed analysis of the results is described below.

#### 2.2.1. Effect on Radial Growth

**Figure 2:**
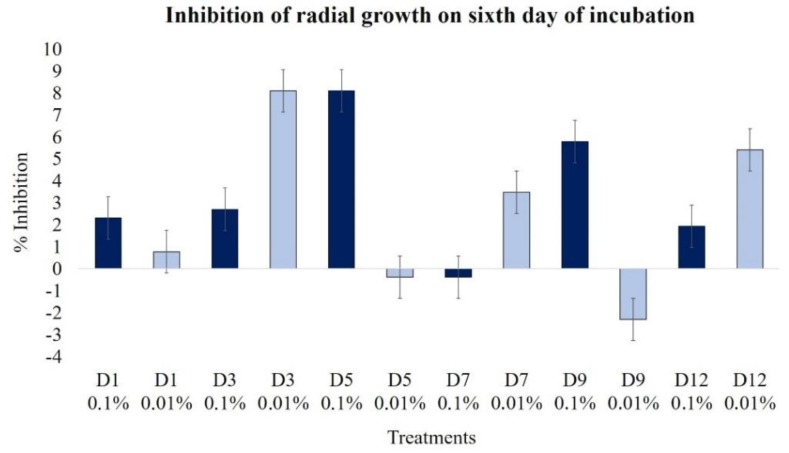
Effects of extracts at 0.1% (dark blue bars) and 0.01% (light blue bars) in the radial growth of *B. cinerea* on the sixth day. Extracts were applied 24 h after conidia inoculation (concentration of 1 × 10^5^ conidia mL^−1^).

The effect of the extracts obtained after several days of fermentation was determined on the sixth day of growth; overall, the effect was not powerful. Extracts D3 and D5, at 0.01% and 0.1%, respectively, were the most inhibitory (near 8%). For the 0.1% extracts, we observed a decreasing inhibition tendency with the oldest extracts, but the less concentrated extracts showed a low but constant inhibition (Figure 2).

#### 2.2.2. Effect on Conidia Germination

**Figure 3:**
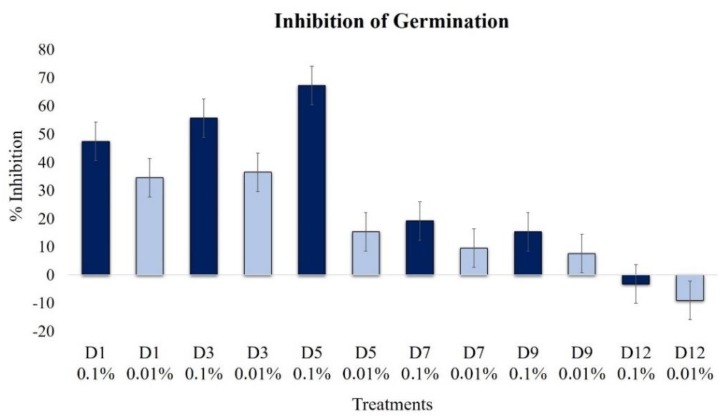
Effects of extracts at 0.1% (dark blue bars) and 0.01% (light blue bars) on the germination of conidia of *B. cinerea* (1 × 10^5^ conidia mL^−1^ at 12 h, 100 rpm, 24 °C).

A significant conidia germination inhibition of *B. cinerea* was achieved during the first five day extracts at 0.1% (Figure 3), with a maximum peak in the D5 extract (67%); then, a decreasing effect was observed. A similar, but less significant, profile was detected at a low concentration of 0.01%. A slight induction of germination was noticed on day 12.

#### 2.2.3. Effect on Elongation of Germ Tubes 

**Figure 4:**
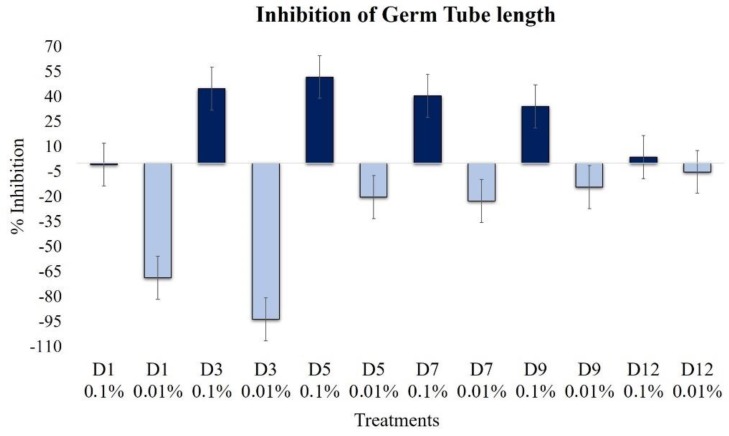
Effects of extracts at 0.1% (dark blue bars) and 0.01% (light blue bars) on the length of germ tubes of *B. cinerea* (1 × 10^5^ conidia mL^−1^, 14 h incubation, 100 rpm, 24 °C).

The effect on the elongation of germ tubes was the opposite for the two analyzed concentrations. The highest concentration of extracts, 0.1%, produced increasing inhibition until the D9 extract, and the maximum effect was produced with the D5 extract (Figure 4). Extracts of D3, D5, and D7 inhibited the elongation by 45%, 52%, and 40%, respectively; similarly, the D9 extract retained activity, presenting an inhibition of 34%.

However, at a lower concentration, i.e., 0.01%, extracts D1 and D3 strongly promoted the elongation of germ tubes, increasing the length by almost 100%, in regards to the control. The extracts from the more advanced fermentation, such as the D12 extracts, did not show significant differences (*P* > 0.05). 

#### 2.2.4. Effect on Pellet Formation and Filamentation

**Figure 5:**
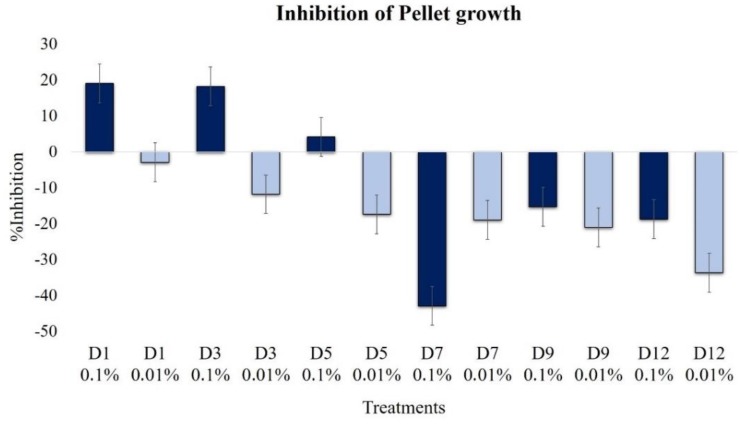
Effects of extracts at 0.1% (dark blue bars) and 0.01% (light blue bars) on *B. cinerea* pellet growth (1 × 10^4^ conidia mL^−1^, 30 h incubation, 100 rpm, 24 °C, light 12 h). The pellet diameters did not show a normal distribution; therefore, nonparametric Kruskal–Wallis analysis was performed. Data on the size of the filamentous region of pellets were subjected to arithmetic transformation with the square root function for the comparison of means.

The highest inhibitions of pellet formation were observed in D1 and D3 extracts at a 0.1% concentration, which was close to 20% (Figure 5), again demonstrating a fungicidal effect. This effect disappeared as the fermentation progressed, especially from the D7 extract and those that followed, which presented a clear promoter profile. At the concentration of 0.01%, all the extracts were inducers of pellet formation, increasing the effect as the fermentation progressed. Thus, the oldest extract, D12, was the most active pellet formation promoter.

**Figure 6:**
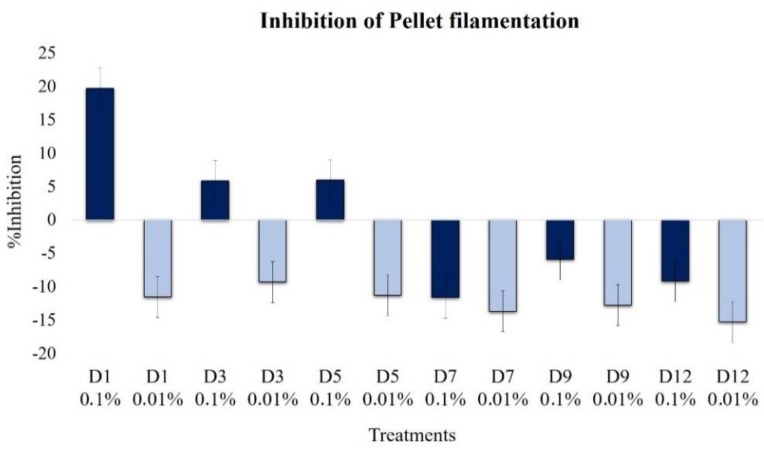
Effects of extracts at 0.1% (dark blue bars) and 0.01% (light blue bars) on *B. cinerea* pellet filamentation (1 × 10^4^ conidia mL^−1^, 30 h incubation, 100 rpm, 24 °C, light 12 h).

Overall, the effect on filamentation followed a similar trend to the pellet formation, with an inhibitory effect of D1–D5 extracts and a slight promoting effect with D7–D12 extracts, all at 0.1% (Figure 6). In contrast, at 0.01%, all extracts induced the growth of the filamentous region by up to 15%.

#### 2.2.5. Effect on Sporulation

**Figure 7:**
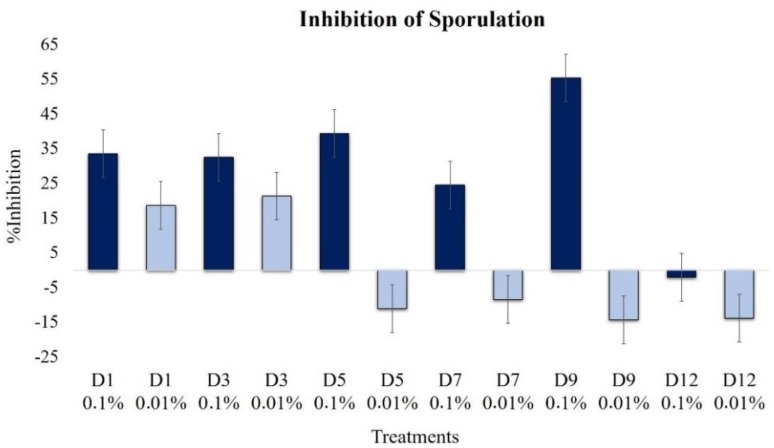
Effects of extracts at 0.1% (dark blue bars) and 0.01% (light blue bars) on sporulation of *B. cinerea* (1 × 10^4^ conidia mL^−1^, 15 days of incubation, 24 °C, light 12 h).

We observed significant changes in sporulation with the application of the extracts, with the effect being dependent on the concentration and the advancement of fermentation (Figure 7). With the application of a high concentration of extracts (0.1%), a strong inhibitory effect was detected, especially with the D9 extract, which reduced the production of conidia by 55.3%, in comparison to the control. 

At the lower concentration, 0.01%, the extracts of the first days of fermentation, D1 and D3, also inhibited the sporulation of the fungus by up to 21.3%. However, extracts from more than five days fermentation were promoters of sporulation.

#### 2.2.6. Effect on Biofilm Formation

**Figure 8:**
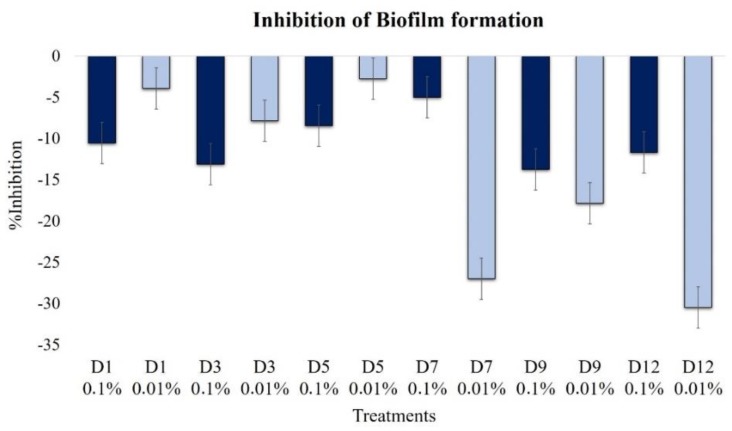
Effects of extracts at 0.1% (dark blue bars) and 0.01% (light blue bars) on *B. cinerea* biofilm formation (1 × 10^4^ conidia mL^−1^, 30 h incubation, 100 rpm, 24 °C, light 12 h).

None of the extracts inhibited the formation of biofilms, and in contrast, all of them induced their formation (Figure 8). However, at the low concentration (0.01%), the oldest extracts, D7–D12, were powerful inducers of biofilm formation, i.e., up to 30.5%.

At a high extract concentration, 0.1%, biofilm formation induction was less than 15%, but minimal levels were observed in D5 and D7 extracts. 

### 2.3. Extract Phytotoxicity

The phytotoxicity of the extracts was evaluated at a 1% concentration in detached leaves of *Solanum lycopersicum* and was estimated using parameters for incidence (number of lesions) and severity (affected area vs. total area). At concentrations of 0.1% and 0.01%, no phytotoxicity was demonstrated, so they were not considered for analysis. 

**Figure 9:**
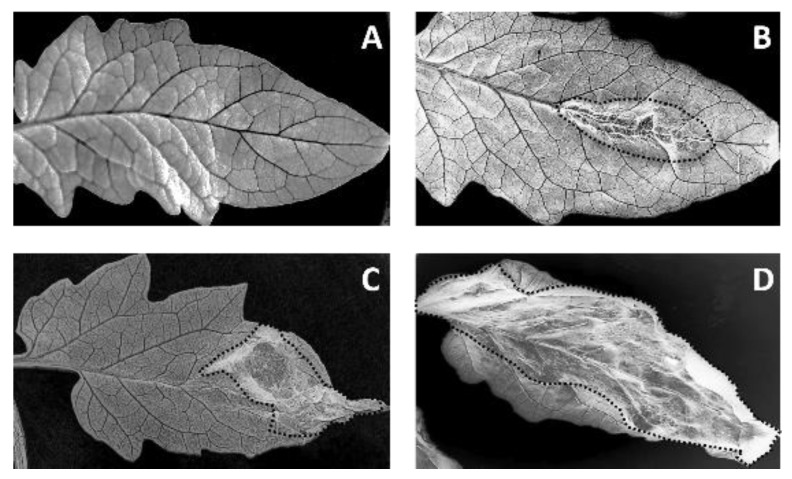
The general appearance of lesions caused by the extracts: (**A**) uninjured leaf, and leaves with (**B**) mild, (**C**) moderate, and (**D**) severe injuries. Black dotted lines indicate the area of the injury.

The phytotoxicity was classified arbitrarily on a fourth scale: Null (-), low (*), moderate (**), and high (***); Figure 9 depicts some examples of injuries caused by the application of extracts. As the control for phytotoxicity, the sesquiterpene botrydial (10 ppm, 32.22 µmol/L) was used.

**Figure 10:**
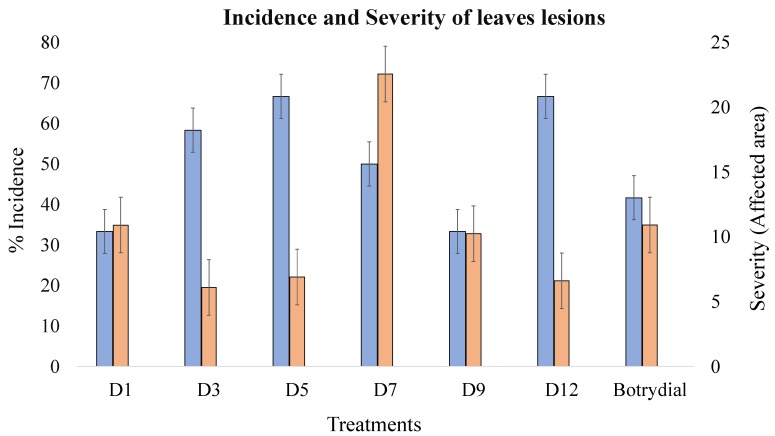
Incidence (blue bars, left Y-axis) and severity (orange bars, right Y-axis) of lesions in Solanum lycopersicum leaves caused by *B. cinerea* extracts.

The incidence of lesions was very high, even with the extracts from the first days of fermentation and increased to a maximum with the D5 extract (66.7%) (Table 4, Figure 10). Subsequently, we observed a slight reduction in D7 and D9 and then another increase with the older extract, D12. Almost all extracts caused a higher incidence than botrydial. Comparatively, we observed an increased incidence up to D5, concomitant with botrydial synthesis. 

For severity (Figure 10), we found no clear correlation with the incidence. For phytotoxicity, almost all extracts showed a minor effect or botrydial control (10.9%). The maximum severity was achieved with D7 extract (22.6%), which was almost double that of the control.

## 3. Discussion

In the search for new methods to control *B. cinerea*, we previously evaluated the effect of several compounds on its growth. The results suggested a modulatory mechanism mediated by *QS* [12], which is a microbial communication mechanism extensively studied in bacteria, although it has now been reported in some yeasts and filamentous fungi [5]. Fungal development shows behavior with a high resemblance to the *QS* of bacteria, as they undergo a process of colonization, followed by pathogenesis, then there is virulence through the synthesis of toxins among other compounds, and finally, there is resistance by sporulation and the formation of a biofilm. Therefore, *B. cinerea* likely synthesizes molecules to modulate its action on host plants dependent on their state of development and nutritional and environmental conditions. For this reason, in this work, we studied the effect of extracts on *B. cinerea,* obtained at different fermentation times on their own cultures, to determine changes in their growth and behavioral development, as well as whether these could be correlated with *QS*. To achieve this goal, cultures were stressed using a medium with low nutrient levels. 

In regards to the influence of the concentration on *QS*, an opposite effect was observed; at high concentrations, inhibition sometimes occurred, whereas at low concentrations, we observed induction in the microbial activity; this is considered to be one of the typical indicators of a *QS* effect [6,9,13]. We observed exactly this behavior in this work by applying extracts of different fermentation durations of *B. cinerea* to new cultures of the same fungus. We found differential and concentration-dependent effects on conidia germination, germ tube growth, pellet-filamentation formation, biofilm formation, and sporulation. In *Aspergillus flavus*, for example, a similar phenomenon has been reported: In the presence of high concentrations of the *QS* compound, phenylethanol, the growth of the fungus was inhibited, but at low concentration, fungus growth is promoted and it inhibited the production of aflatoxins [14]. The same was detected for farnesol, which inhibits growth in the producer fungi (*Candida albicans*) at high concentrations, but at low concentrations, regulates processes related to morphology and virulence [9,15].

However, The UHPLC/MS profile (Supplementary Material) was not the typically expected profile in the fermentation of this fungus, since the culture was subjected to stress conditions to strengthen *QS*-type behavior using a nutrient-poor medium (Sabouraud broth at 50%). Through HPLC/MS, we monitored the presence of several secondary metabolites in the *Botrytis* extracts. Thus, the presence of the botrydial toxin was detected in early cultures, but its biosynthesis was only maintained until D7. Two aromatic compounds, 1-phenylethanol and 3-phenyl-1-propanol, were biosynthesized from D5 until the end of the fermentation. The botryendial and eremophil-9-ene-1,11-diol compounds were produced in the late stages of fermentation. The compound 1-phenylethanol was reported to be a QS modulator in *Saccharomyces cerevisiae* [9], whereas 3-phenyl-1-propanol was produced in matsutake and shitake fungi [16,17]. 

The most important effects related to the inhibition of conidia germination, germ tube growth, pellets, and sporulation indicate the production of related biosynthesis molecules such as botrydial, botrydial, and dihydrobotrydial. This type of compound has been reported as toxins in extracts of *B. cinerea* from the third day of incubation [18]. The first one is perhaps the most significant and abundant phytotoxin produced by *B. cinerea* because this compound induces chlorosis and cell collapse [19]. Similar to the results obtained by Durán-Patrón [20], in this work, we detected the presence of botrydial from D3 until D7. Low botrydial concentrations seem to play an essential role in the life cycle of the fungus; at high concentrations, the compound is autoinhibitory [20]. Other toxins were also found to be botryendial (D7–D12), which could explain the greater damage severity caused by the extracts in comparison to pure botrydial. 

Botryendial and eremophil-9-ene-1,11-diol, which were synthesized from day 7, seem to be involved in other processes related to pellet aggregation, sporulation, and biofilm formation. The presence of these compounds agrees with 1-phenylethanol and 3-phenylpropanol, molecules that have previously been described as *QS* self-inducers in yeasts and fungi. However, it is not possible to define which of the two classes of molecules, terpenes or phenyl-derivatives, is responsible for the *QS*-type response. 

The concentration of conidia, light, pH, nitrogen source, and carbon affects the germination of *B. cinerea* [21,22]. In this work, the level of germination inhibition was dependent on the concentration of the extracts. Thus, the D5 extract at 0.1% produced the highest inhibition (above 60%) and matched the production of botrydial. The D1 and D3 extracts also showed high germination inhibition, suggesting an independent effect of toxin production, similar to the *QS* process [17]. The inhibitory effect on conidia germination of the D5 extract matched the maximum production of the phytotoxin botrydial, which was reported by other researchers on the fifth day of cultivation [20], and presented self-inhibiting effects on the growth of *B. cinerea*.

The activity of the extracts on the elongation of germ tubes depended on the concentration and fermentation time. At a concentration of 0.01%, for example, D1, D3, and D5 extracts promoted the development of germ tubes, but also strongly inhibited germination, confirming the presence of compounds that act as differential inductors or repressors of growth [23]. 

Conidia aggregation and subsequent pellet formation depend on factors such as the concentration of the inoculum, the composition of the culture medium, the pH, and the genetic characteristics of the strain, among others [24]. In this work, we observed that changes in the size and filamentation of pellets by exposure to extracts are similar to the *QS* mechanism reported in *C. albicans* mediated by farnesol–tyrosol. When these compounds were added at specific stages of growth, they induce or suppress cell aggregation [25]. If this mechanism is also present in *B. cinerea*, the elucidation and understanding of the mechanism could be exploited to disrupt the capacity of hyphal aggregation and decrease the infective potential in plant tissue. The fungi *Aspergillus nidulans, Aspergillus terreus, Penicillium chrysogenum*, and *Penicillium sclerotiorum* synthesize oxylipins and lactones that, when exogenously supplemented, produce changes in the production of toxins and sporulation without affecting their growth [11,26]. The inhibition of sporulation was most evidently detected at the highest c extract concentration (0.1%); the maximum value was observed with the extract D9 (55.3%). At low concentrations, we observed inhibition with D1 and D3, but with D5–D12, the effect was clearly the induction of sporulation.

The detection of eremophil-9-ene-1,11-diol in D12 extract at 0.1% correlates with the absence of inhibition in sporulation, although at 0.01%, a sporogenic effect was observed. These results also agree with those reported by Pinedo about the significant increases in the sporulation of two strains of *B. cinerea* (UCA992 and B05.10) when they were exposed to the pure compound [27]. However, the biosynthetic sequence botrydiol, botrydial, botryendial, and dihydrobotrydial is well correlated to several observed symptoms and could suggest a previous *QS* effect independent of the changes in spores due to the high virulence of the botrydial.

The formation of biofilm occurs in response to self-induced signals, which depend on environmental, nutritional, or chemical adverse conditions [13]. The effects of the extract on *B. cinerea* showed a high tendency to induce the formation of biofilm at 0.1%, especially in the first and the last stages of fermentation. However, at the low concentration, 0.01%, the biofilm inductor effect was only observed from the seventh day of fermentation, as was the case for sporulation [7].

We observed no comparable behavior between the incidence and the severity of extracts of *B. cinerea* in *S. lycopersicum*. We found an increasing incidence up to D5, concomitant with botrydial synthesis, and subsequently, another increase in D12, for which botryendial and eremophil-9-ene-1,11-diol compounds could be responsible, since the synthesis of these compounds started on the ninth day. The effect of the D12 extract was above that of the control botrydial (41.7%), which could be explained by a possible collaboration between the toxins or their active degradation substances. A different trend was observed during the estimation of the severity since the most significant effect was obtained with the D7 extract (22.6%), which was double that of the control botrydial (10.9%), but it also produced a high incidence of 50%. Likewise, phytotoxicity was evidenced in all extracts, above the levels produced by the botrydial control. D5 increased the incidence to 60%, in comparison to the control. However, with two extracts, D1 and D9, a lower effect was produced.

From a general point of view, these toxins are the virulence tools of *B. cinerea,* but we do not exclude the possibility that they may also act as modulators of *QS*. In strains of *B. cinerea* submitted to chemical stress, a *QS* effect was reported with several sporogenic eremophil-9-ene-1,11-diol derivatives in cultures after more than three weeks of fermentation [28]. The regulation of gene expression in *Trichoderma arundinaceum* by botrydial and botcinins has been previously established in a process named by the authors as “a metabolic dialogue”, but, from a biochemical point of view, can be considered as a *QS* effect [29]. Even some mycotoxins seem to play a role more related to *QS* than to animal cells, as has been shown in *Fusarium* [30,31]. However, the effect of the self-inducing substances involved in *QS* does not only occur at the intra or inter-bacterial level, as one of the signaling lactones has also been found to modulate the chemical defense mechanisms of plants, especially at the level of the jasmonate pathway [32].

In short, this paper provides evidence on the presence of molecules in *B. cinerea* extracts that are involved in the modulation of growth, development, virulence, and resistance like *QS* in bacteria. Purification and identification of these molecules will be the subject of future work. The existence of *QS* in *B. cinerea* provides new possibilities for the non-biocidal control of pathogenic filamentous fungi and the understanding of their biochemistry and its harmful effects. Thus, new compounds could inhibit the virulence of *B. cinerea* through the inhibition of the production of phytotoxins, and even resistance, inhibiting the formation of biofilm.

## 4. Materials and Methods

### 4.1. Equipment and Software

Crops were submerged in a three-level orbital agitation shaker (HD-4000, Actum, Medellín, Colombia), with fluorescent tube lamps (15W-T8, 26 mm, 6500 K). Cultures in solid media were grown in a conventional natural convection incubator (WTB Binder, Tuttlingen, Germany). 

The length of germ tubes, diameters, and pellet filamentation (Figure 10) were measured via digital image analysis with ImageJ software (National Institutes of Health, Bethesda, Maryland. USA). The micrographs were obtained with a digital sensor (16 MP, f/1.8, 28 mm. Lg Electronics, Yeonji-dong, Busan, South Korea), coupled to a conventional optical microscope (Alphaphot-2 YS2, Nikon, Tokio, Japan) [33,34].

The data obtained were stored in the Microsoft Excel 2016 office suite (Microsoft, Redmond, Washington, US) and processed through Statgraphics Centurion XVI Version 16.2.04 (Statgraphics Technologies Inc. The Plains, Virginia, US) and SPSS Statistics V23.0 (IBM, Armonk, Nueva York, US).

### 4.2. Fungal Strain

*B. cinerea* was isolated from infected Chonto tomato variety fruits (*S. lycopersicum* L.). Subsequently, monosporic cultures were grown on dextrose potato agar (PDA; Merck, Darmstadt, Germany) and were incubated until the profuse sporulation of the fungus (24 °C, photoperiod 12 h).

### 4.3. Molecular Identification

The molecular identification of the strain was performed at the National Center for Genomic Sequencing, Universidad de Antioquia (Medellin, Colombia). Briefly, the DNA was extracted, and we conducted sequencing and phylogenetic analysis of the ribosomal Internal Transcribed Spacer (ITS) markers, ITS1 and ITS2, and the amplification of the *hsp60* and *rpb2* genes. 

### 4.4. Obtention and Analysis of B. cinerea Extracts

The conidia of a 12-day culture were obtained by adding 20 mL of sterile distilled water and surface scraping. The suspension was filtered into sterile gauze sieve and inoculated in 500 mL Erlenmeyer’s flask with 300 mL of Sabouraud Broth 50% (Merck, Darmstadt, Germany) (1 × 10^5^ conidia/mL). The media were incubated for 12 days (100 rpm, 24 °C, photoperiod 12 h). 

On days 1, 3, 5, 7, 9, and 12, the culture supernatant recovered by centrifugation and filtration before freeze-drying; 50 g of lyophilized medium was extracted four times with 300 mL of ethyl acetate, dehydrated with Na_2_SO_4_, and concentrated under vacuum to dryness and finally were stored at −20 °C until used [35]. For bioassays, the extracts were solubilized in methanol and evaluated at concentrations of 0.1% and 0.01% (% p/v). Each extract will be described as D1, D3, D5, D7, D9, and D12, corresponding to the number of days of fermentation.

We searched for toxins and other compounds in the extracts using high-performance liquid chromatography–quadrupole-time of flight mass spectrometry (HPLC-QTOF) analyses. The elution gradient was formed in a C-18 column (Acclaim C18; 100 mm; diameter, 2.1 mm; particle size, 1.5 mm; Thermo Fisher Scientific, Waltham, Massachusetts, US). The C-18 column was eluted with a gradient of (A) 0.1% formic acid in acetonitrile and (B) 0.1% formic acid in water. Chromatographic elution gradients started with 90% solvent A; in 20 min, solvent B increased to 60% and was maintained for 5 min, followed by an increase to 100% in a 5 min interval. These conditions were maintained for five more minutes, and then the system returned to the initial solvent composition and was stabilized for 5 min. The flow rate was 300 L/min, with an injection volume of 10 L; the temperature of the column was maintained at 40 °C and the self-sampler at 10 °C.

The high-resolution mass spectra were obtained using Ultra-High Resolution Qq-Time-Of-Flight (Bruker Impact II UHR–QqTOF equipment; Billerica, Massachusetts, US) with an electrospray ionization (ESI) source. The gas in the cone and the dissolving gas was dry nitrogen. The flow of the desolvation gas was optimized to approximately 8 L/min. Other adjusted parameters were: Capillary voltage, 4500 V in positive ionization mode; source temperature, 200 °C; and collision energy, 5 eV. The mass range was from *m/z* 50 to 1200 in scan mode (Auto MS/MS).

### 4.5. Effect of Extracts on B. cinerea Growth

#### 4.5.1. Effect on Radial Growth

We inoculated 10 μL of a *B. cinerea* conidia suspension (1 × 10^5^ conidia/mL) in the center of a Petri plate (90 mm) with Sabouraud 2% glucose agar (Merck KGaA, Darmstadt, Germany) and incubated at 24 °C (photoperiod 12 h). After 24 h, 10 μL of the extracts was deposited on the inoculum (0.1% and 0.01%, w/v); from the third day of incubation, the diameter of the colonies was measured every 24 h. Each treatment was evaluated in triplicate. Methanol was used in growth control.

#### 4.5.2. Effect on Germination

In 2 mL vials, Sabouraud broth (30 g/L), extracts (0.1% and 0.01%) were added to *B. cinerea* (1 × 10^5^ conidia/mL). The mixture was incubated for 12 h (100 rpm, 24 °C) and the germination percentage of the conidia was determined. Germination was considered positive when the length of the germ tube was equal to or greater than the size of the conidia [36]. The growth control contained the same mixture, except for the extract. Each treatment was assessed in triplicate, for a total of 50 units per repetition (*N* = 150).

#### 4.5.3. Effect on Germ Tube Development 

We added the extracts (0.1% and 0.01%) to a suspension of 1 × 10^5^ conidia/mL of *B. cinerea* incubated for 2 h in Sabouraud broth. The suspension was re-incubated for 12 h, and at the end of the period, the lengths of the germ tubes were estimated by digital image analysis (40×) [37]. Treatments were evaluated in triplicate, for a total of 20 units per repetition (*N* = 60).

#### 4.5.4. Effect on pellet formation

**Figure 11:**
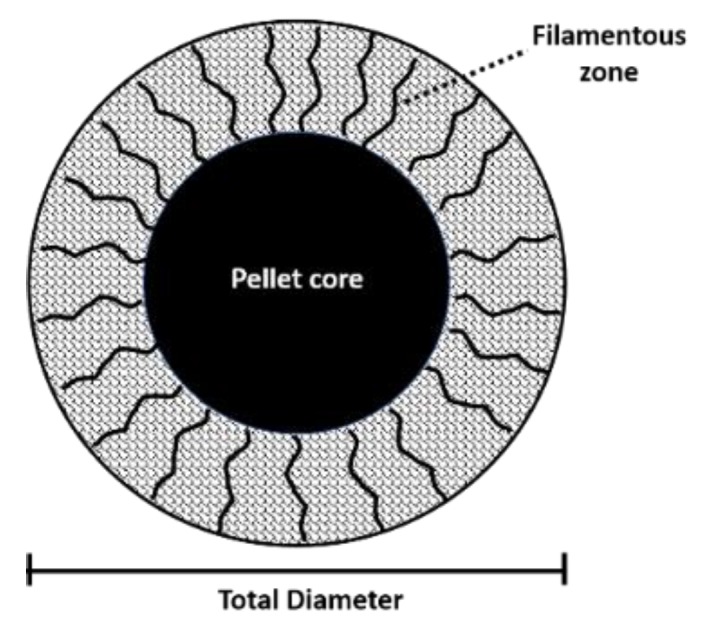
Pellet measurement including core diameter, total diameter, and length of filaments. Measurements were performed using ImageJ software.

We inoculated 50 mL of Sabouraud broth in 500 mL Erlenmeyer bottles with 1 × 10^4^ conidia/mL of *B. cinerea,* which was incubated for six hours (100 rpm, 24 °C,); extracts (0.1% and 0.01%) were subsequently added and incubated again for 24 h, with a photoperiod of 12 h. Finally, the pellets were removed and carefully washed with sterile distilled water to eliminate the remaining culture medium. The pellets were photographed using an optical microscope (4×), and the diameter and filamentation were measured using digital image analysis (Figure 11). Treatments were evaluated by measuring 20 pellets per treatment.

#### 4.5.5. Effect on Biofilm Formation in Submerged Cultures

The methodology described in Section 4.5.4 was used to evaluate biofilm formation, with an incubation time of 72 h after the addition of treatments. After that, the culture medium was removed along with the suspended biomass, and with 100 mL of sterile distilled water, the bottle was carefully washed three times to remove the residues of medium and non-adhesive mycelium. Finally, the fresh weight of the biomass attached to the bottle was determined [38]. Each treatment was conducted in triplicate.

#### 4.5.6. Effect on Sporulation

We inoculated 10 μL of a conidia suspension (1 × 10^5^ conidia/mL) in the center of a Petri plate with Sabouraud agar and incubated for 24 h (24 °C, photoperiod 12 h). Then, 10 μL of the extracts (0.1% and 0.01%, w/v) were deposited on the inoculum of *B. cinerea* and re-incubated for 15 days under the same conditions. The conidia were recovered by superficial scraping, and the concentration was determined with a hematocytometer.

### 4.6. Phytotoxic Effects of the Extracts of B. cinerea

The phytotoxic effect of extracts on detached leaves of Chonto tomato (*Solanum lycopersicum*) was evaluated. The true leaves were detached from the stem, disinfected, and maintained in a wet chamber for six weeks at 21–24 °C with a 14 h fluorescent light/10 h dark photoperiod [39]. Then, 10 µL of the extract were deposited in four equidistant points from the leaves and incubated for seven more days. Each treatment was conducted in triplicate (*N* = 12). Methanol and botrydial (10 ppm = 32.22 µMol) were used as the control; the latter is recognized for its plant phytotoxic effects [40].

At the end of the incubation period, the incidence and severity of the lesions caused by the extracts were determined. The affected areas were estimated via digital image analysis with ImageJ software.

## 5. Conclusions

In this work, we found some evidence of the presence of quorum sensing in the fungus *B. cinerea*, since extracts with different fermentation times produced concentration-dependent effects on the growth and development of the fungus. This communication process is probably achieved through the production of 1-phenylethanol, 3-phenylpropanol, and botrydial and eremophil-9-ene-1,11-diol sesquiterpenes, although more research is needed to confirm this finding.

The presence of *QS* in *B. cinerea* can be analyzed to generate new knowledge to understand its phytopathological behavior and to eventually design other chemical and molecular tools that allow for its non-biocidal control.

## Figures and Tables

**Table 1:** Production of extracts of *Botrytis cinerea* at different growing times.

Time (day)	Name of Extract	Weight (mg)
1	D1	111.5
3	D3	98.9
5	D5	282.9
7	D7	278.5
9	D9	393.9
12	D12	219.6

**Table 2:** Compounds detected in *B. cinerea* extracts according to the time of fermentation.

Type of Compound	Compound	Fermentation Time (days)					
		1	3	5	7	9	12
Terpenes	Botrydial	-	+	+	+	-	-
	Botrydiol	+	-	-	-	-	-
	Dihydrobotrydial	-	+	+	-	-	-
	Botryendial	-	-	-	+	+	+
	Eremophil-9-ene-1α,11-diol	-	-	-	-	+	+
Polyketides	Botcinin D	-	-	+	+	+	+
*QS* Molecules	1-Phenylethanol	-	-	+	+	+	-
	3-Phenyl-1-propanol	-	-	+	+	+	+
	Farnesol	-	-	-	-	-	-
	Tyrosol	-	-	-	-	-	-

Note: +, presence; -, absence. The HPLC/MS profile and spectra are provided in Supplementary Material.

**Table 3:** Inhibitory activity of *B. cinerea* extracts on its growth.

Evaluated Parameter	Percentage of Inhibition (%)											
	*D1*		*D3*		*D5*		*D7*		*D9*		*D12*	
	0.1%	0.01%	0.1%	0.01%	0.1%	0.01%	0.1%	0.01%	0.1%	0.01%	0.1%	0.01%
Radial growth (day 6)	2.3	0.8	2.7	8.1	8.1	−0.4	−0.4	3.5	5.8	−2.3	1.9	5.4
Conidia Germination	47.4	34.6	55.8	36.5	67.3	15.4	19.2	9.6	15.4	7.7	3.2	9.0
Germ tube growth	0.8	68.7	44.8	93.7	51.9	−20.2	40.4	−22.5	34.3	−14.2	3.8	−5.1
Pellet growth	19.1	−2.9	18.2	−11.9	4.1	17.4	−42.9	−19.0	−15.3	−21.1	−18.8	−33.7
Pellet hairy region	19.7	−11.6	5.8	−9.3	−6.0	−11.3	11.4	−13.7	−5.9	−12.8	−9.2	−15.3
Sporulation	33.5	18.6	32.4	21.3	39.4	−11.2	24.5	−8.5	55.3	−14.4	−2.1	−13.8
Biofilm	−10.5	−4.0	−13.1	−7.9	−8.4	−2.7	−5.0	−27.0	−13.7	−17.8	−11.7	−30.5
	**Phytotoxicity (1%)**											
Incidence	20.1		−39.8		−60.0		−19.9		20.1		−60.0	
Severity	−81.1		−3.9		−12.0		−218.1		−131.3		−31.7	

Negative values indicate a promoting effect. Red, a high inhibitory effect; coral, a medium inhibitory effect; light blue, a low inhibition effect; white, a growth-promoting effect.

**Table 4:** Incidence and severity in tomato leaves caused by extracts of *B. cinerea*.

Treatment	Incidence (%)	Average Area of the Lesion (cm^2^)	Severity (%) (Lesion Area/Total Area)	Phytotoxicity
D1	33.3	0.42	10.9	**
D3	58.3	0.24	6.1	*
D5	66.7	0.26	6.9	*
D7	50	0.73	22.6	***
D9	33.3	0.53	10.3	**
D12	66.7	0.30	6.6	*
Botrydial	41.7	0.23	10.9	**
Methanol (control)	0	-	-	-

Observed phytotoxicity: -, null; *, low; **, moderate; ***, high.

## Supplementary Materials

The following are available online at https://www.mdpi.com/2223-7747/9/2/168/s1, Table S1: Supplementary data. LC-MS fragmentation of compounds, Table S2. Supplementary data. Compounds detected at several times (LC-MS), Figure S3. Supplementary data. Mass spectrometry of some detected compounds.
