# Supplementary material for: The Search for Quorum Sensing in Botrytis cinerea: Regulatory Activity of Its Extracts on Its Development
Source: Plants (Basel). 2020 Jan 31;9(2):168. doi: 10.3390/plants9020168 (PMC7076370; doi:10.3390/plants9020168)

Table S1. Supplementary data. LC-MS fragmentation of compounds.

Name	Formula	Retention time (RT) (min)	Calculated Mass (*m/z*)	Theoretical Mass (m/z)	Adduct	Mass Error (ppm)	MS² fragmentation
1-phenylethanol	$C_8H_{12}O$	7,3-8,3	123,0804	123,0804	$[M + H]^+$	0	105.7597, 95.0504, 81.0661, 79.0595, 67.0545, 65.0396, 56.0495, 53.0381, 51.0224, 44.9794
Botrydial	$C_{17}H_{27}O_5$	5,3-5,4	311,1855	311,1853	$[M + H]^+$	0,6	263.1680, 233.1554, 215.1440, 187.1459, 173.1325, 145.1012, 119.0843, 105.0702, 93.0687, 79.0549
Dihidrobotrydial	$C_{17}H_{28}O_5Na$	1,8	335,1824	335,1828	[M+Na]+	-1,2	315.0316, 296.0480, 275.1578, 260.0097, 218.1647, 187.9591, 167.1456, 154.0685, 119.0834, 103.0527, 85.1007, 55.0581, 45.0326
Botryendial	$C_{17}H_{25}O_4$	6,5-7,3	293,1749	293,1747	$[M + H]^+$	0,7	292.2002, 264.0953, 234.1836, 215.1410, 206.1264, 187.1463, 171.1151, 159.1170, 145.0997, 130.0752, 117.0661, 105.0695, 79.0540, 67.0532, 61.0267, 55.0530
Botcinin D	$C_{20}H_{31}O_6$	9,1-9,2	367,2117	367,2115	$[M + H]^+$	0,5	225.1121, 179.1064, 169.0861, 151.0751, 137.0962, 125.0955, 109.0644, 67.0544, 55.0544
4-eremophil-9-ene-1α,11-diol	$C_{15}H_{27}O_2$	7,4-7,5	239,2006	239,2006	$[M + H]^+$	0,0	239.1640, 239.1199
3-phenyl-1-propanol	$C_9H_{13}O$	7,9-8,2	137,0953	137,096	$[M + H]^+$	-5,1	136.0803
Botrydiol	$C_{17}H_{30}O_5$	7,1	315,2166	315,2166	$[M + H]^+$	0,0	312.1587, 295.1543, 266.1166, 253.1417, 238.1225, 219.1374, 201.1267, 194.1171, 173.1319, 135.0789, 120.0801, 91.0539

Table S2. Supplementary data. Compounds detected at several times (LC-MS).

Type of Compound	Compounds	Formula	Extract day 1			Extract day 3			Extract day 5			Extract day 7			Extract day 9			Extract day 12		
			Calculated Mass (*m/z*)	Theoretical Mass (m/z)	Mass Error (ppm)	Calculated Mass (*m/z*)	Theoretical Mass (m/z)	Mass Error (ppm)	Calculated Mass (*m/z*)	Theoretical Mass (m/z)	Mass Error (ppm)	Calculated Mass (*m/z*)	Theoretical Mass (m/z)	Mass Error (ppm)	Calculated Mass (*m/z*)	Theoretical Mass (m/z)	Mass Error (ppm)	Calculated Mass (*m/z*)	Theoretical Mass (m/z)	Mass Error (ppm)
Terpenes	Botrydial	$C_{17}H_{27}O_5$				311,1858	311,1853	-1,61	311,1852	311,1853	0,32	311,1857	311,1853	-1,29						
	Botrydiol	$C_{17}H_{30}O_5$	337,1985	337,1985	0															
	Dihidrobotrydial	$C_{17}H_{28}O_5Na$				335,1834	335,1828	-1,79	335,1828	335,1829	0,30									
	Botryendial	$C_{17}H_{25}O_4$										293,1746	293,1747	0,34	293,174	293,1747	-2,39	293,174	293,1747	2,39
	eremophil-9-ene-1α,11-diol	$C_{15}H_{27}O_2$													239,2008	239,2006	0,84	239,2007	239,2006	-0,42
Polyketides	Botcinin D	$C_{20}H_{31}O_6$							367,2112	367,2115	-0,82	367,2109	367,2115	-1,63	367,211	367,2115	-1,36	367,211	367,2115	1,36
QS Molecules	1-Phenylethanol	$C_8H_{12}O$							123,0809	123,0804	4,06	123,0808	123,0804	3,25	123,0808	123,0804	3,25			
	3-Phenyl-1-propanol	$C_9H_{13}O$							137,0968	137,0961	5,11	137,0966	137,0961	3,65	137,0967	137,0961	4,38	137,0965	137,0961	2,92

Figure S3. Mass spectrometry of some detected compounds

1-phenylethanol

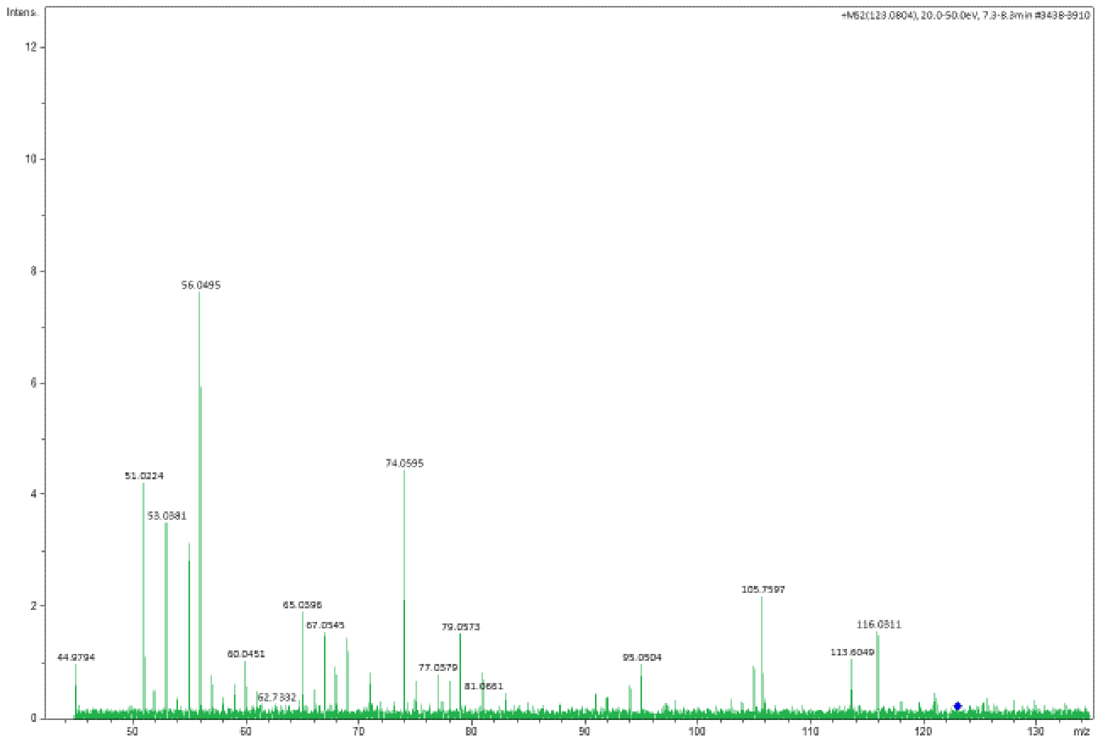

Botrydial

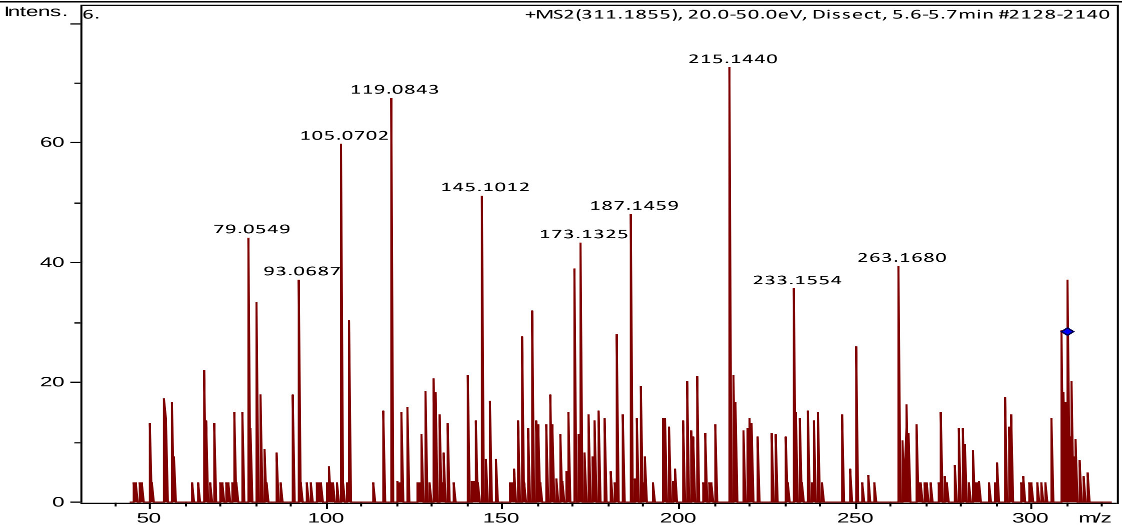

Dihidrobotrydial

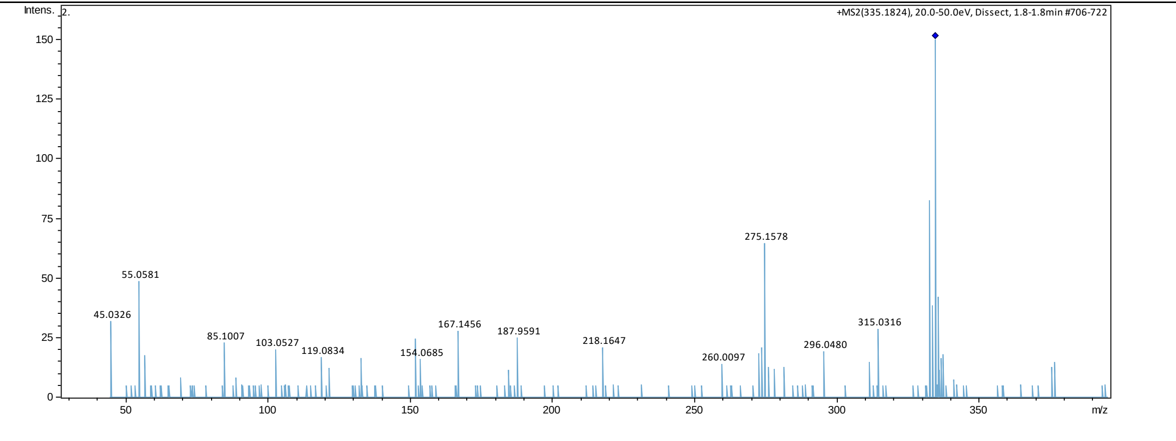

Botryendial

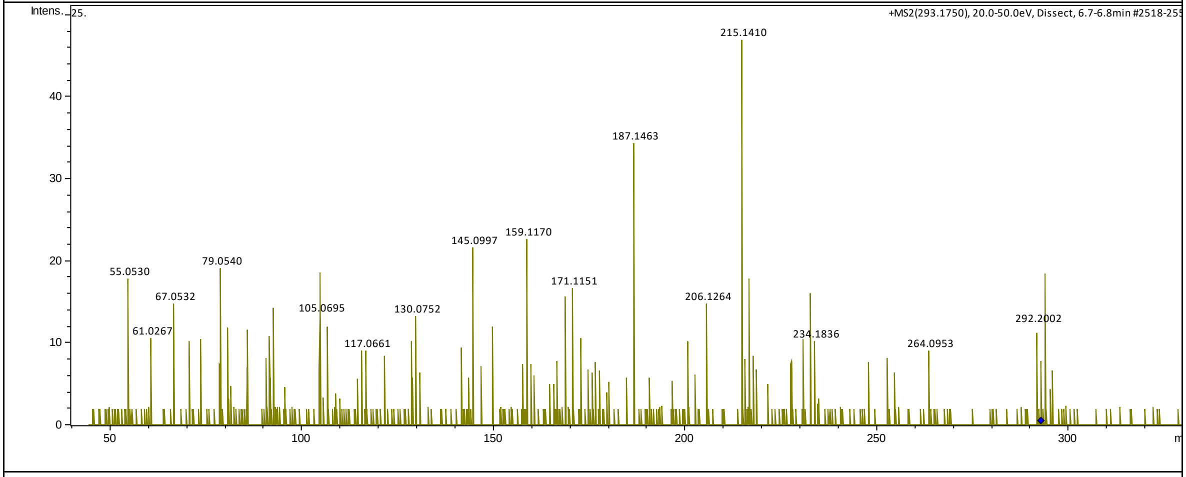

Botcinid D

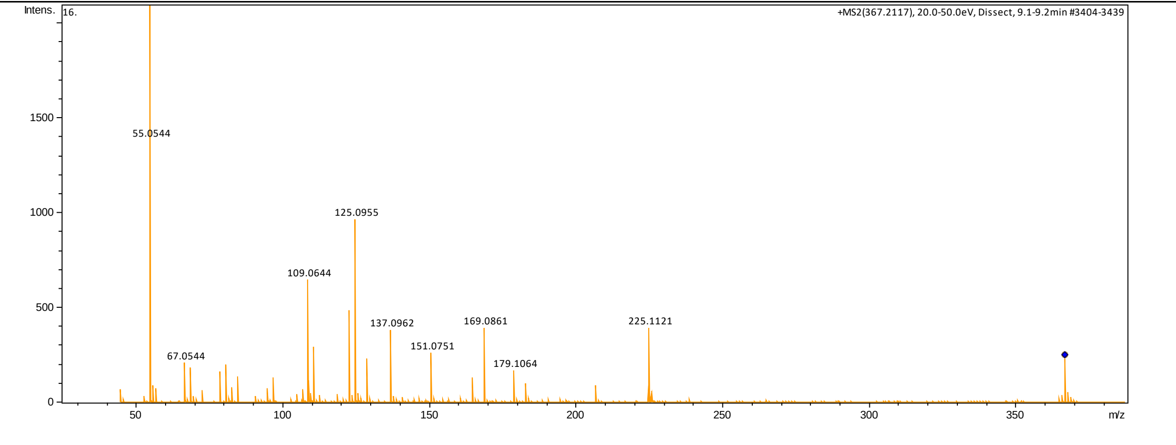

Eremophil-9-ene-1,11-diol

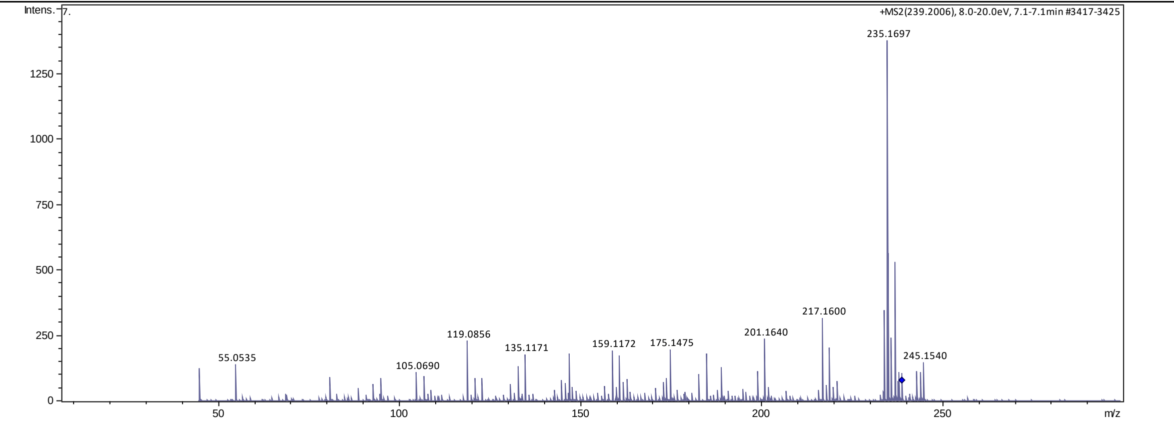

3-phenyl-1-propanol

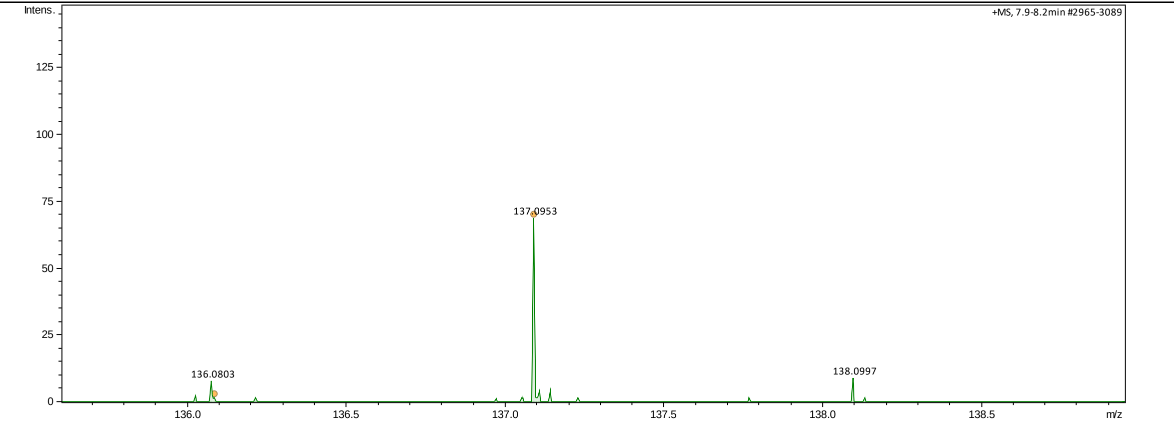

Botrydiol

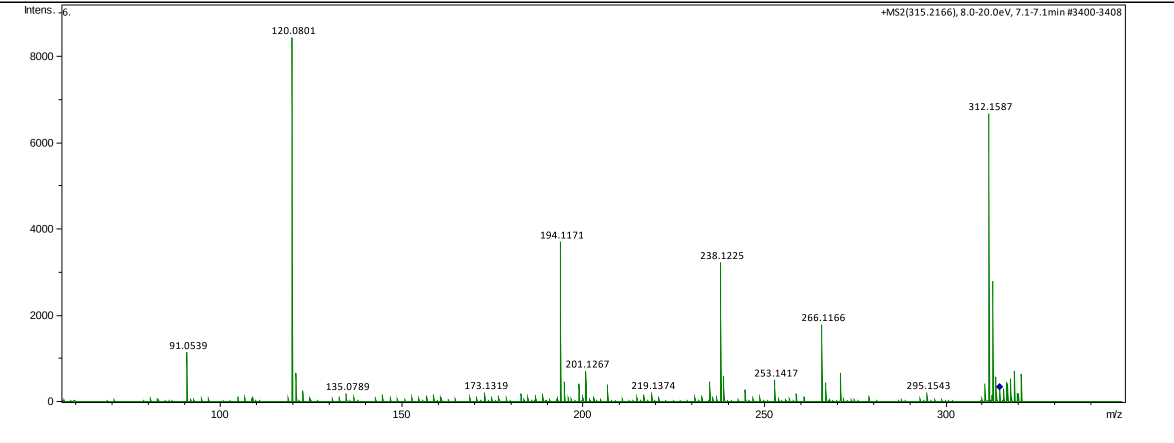

Supplement: Supplementary file 1 [file plants-09-00168-s001.pdf]
